# Myasthenia gravis seronegative for acetylcholine receptor antibodies in South Korea: Autoantibody profiles and clinical features

**DOI:** 10.1371/journal.pone.0193723

**Published:** 2018-03-08

**Authors:** Kee Hong Park, Patrick Waters, Mark Woodhall, Bethan Lang, Thomas Smith, Jung-Joon Sung, Kwang-Kuk Kim, Young-Min Lim, Jee-Eun Kim, Byung-Jo Kim, Jin-Sung Park, Jeong-Geon Lim, Dae-Seong Kim, Ohyun Kwon, Eun Hee Sohn, Jong Seok Bae, Byung-Nam Yoon, Nam-Hee Kim, Suk-Won Ahn, Jeeyoung Oh, Hyung Jun Park, Kyong Jin Shin, Yoon-Ho Hong

**Affiliations:** 1 Department of Neurology, Gyeongsang National University Hospital, Jinju, Republic of Korea; 2 Nuffield Department of Clinical Neurosciences, John Radcliffe Hospital, Oxford, United Kingdom; 3 Department of Neurology, Seoul National University Hospital, Seoul, Republic of Korea; 4 Department of Neurology, Asan Medical Center, University of Ulsan College of Medicine, Seoul, Republic of Korea; 5 Department of Neurology, Seoul Medical Center, Seoul, Republic of Korea; 6 Department of Neurology, Korea University College of Medicine, Korea University Anam Hospital, Seoul, Republic of Korea; 7 Department of Neurology, Kyungpook National University, School of Medicine, Daegu, Republic of Korea; 8 Department of Neurology, Keimyung University School of Medicine, Daegu, Republic of Korea; 9 Department of Neurology, Pusan National University Yangsan Hospital, Yangsan, Republic of Korea; 10 Department of Neurology, School of Medicine, Eulji University, Seoul, Republic of Korea; 11 Department of Neurology, Chungnam National University Hospital, Daejeon, Republic of Korea; 12 Department of Neurology, College of Medicine, Hallym University, Seoul, Republic of Korea; 13 Department of Neurology, Inha University Hospital, Incheon, Republic of Korea; 14 Department of Neurology, Dongguk University Ilsan Hospital, Goyangsi, Gyeonggido, Republic of Korea; 15 Department of Neurology, Chung-Ang University Hospital, Chung-Ang University College of Medicine, Seoul, Republic of Korea; 16 Department of Neurology, Konkuk University Medical Center, Seoul, Republic of Korea; 17 Department of Neurology, Mokdong Hospital, Ewha Womans University School of Medicine, Seoul, Republic of Korea; 18 Department of Neurology, Haeundae Paik Hospital, Inje University College of Medicine, Busan, Republic of Korea; 19 Department of Neurology, Seoul National University College of Medicine, Seoul National University Seoul Metropolitan Government Boramae Medical Center, Seoul National University Medical Research Council, Seoul, Republic of Korea; Case Western Reserve University Jack Joseph and Morton Mandel School of Applied Social Sciences, UNITED STATES

## Abstract

Acquired myasthenia gravis (MG) is a prototype autoimmune disease of the neuromuscular junction, caused in most patients by autoantibodies to the muscle nicotinic acetylcholine receptor (AChR). There seem to be ethnic and regional differences in the frequency and clinical features of MG seronegative for the AChR antibody. This study aimed to describe the autoantibody profiles and clinical features of Korean patients with generalized MG seronegative for the AChR antibody. A total of 62 patients with a high index of clinical suspicion of seronegative generalized MG were identified from 18 centers, and we examined their sera for antibodies to clustered AChR, muscle-specific tyrosine kinase (MuSK), and low-density lipoprotein receptor-related protein 4 (LRP4) by cell-based assays (CBA) and to MuSK by radioimmunoprecipitation assay (RIPA). We also included 8 patients with ocular MG, 3 with Lambert-Eaton myasthenic syndrome, 5 with motor neuron disease, and 9 with other diagnoses as comparators for the serological testing. Antibodies were identified in 25/62 (40.3%) patients: 7 had antibodies to clustered AChR, 17 to MuSK, and 2 to LRP4. Three patients were double seropositive: 1 for MuSK and LRP4, and 2 for MuSK and clustered AChR. The patients with MuSK antibodies were mostly female (88.2%) and characterized by predominantly bulbar involvement (70%) and frequent myasthenic crises (58.3%). The patients with antibodies to clustered AChR, including 2 with ocular MG, tended to have a mild phenotype and good prognosis.

## Introduction

Acquired myasthenia gravis (MG) is an autoimmune disease of the neuromuscular junction, characterized by exertional weakness and fatigability [1]. It is caused in most patients by autoantibodies to the muscle nicotinic acetylcholine receptor (AChR), but the antibodies are not detected on conventional radioimmunoprecipitation assay (RIPA) in 20% of patients with generalized MG and 50% with ocular MG [2]. A cell-based assay (CBA) was established to detect low-affinity antibodies binding to clustered AChR expressed on the cell membrane in a more native state [3]. The CBA for clustered AChR antibody has been shown to be specific and positive in 16% to 60% of RIPA-negative patients [3–5]. Patients with antibodies only to clustered AChR reportedly tend to develop the disease earlier, with frequent prepubertal onset, and to have a mild phenotype with high prevalence of ocular MG [6]. Autoantibodies to muscle-specific tyrosine kinase (MuSK) have been reported in approximately 5% of patients with generalized MG with distinctive and even atypical clinical features, such as predominant bulbar and respiratory muscle weakness and marked muscle atrophy [7]. MuSK antibodies interfere with AChR clustering through the activity of IgG4 autoantibodies, rather than through complement-mediated damage by AChR antibodies [8]. Recently, autoantibodies to low-density lipoprotein receptor-related protein 4 (LRP4) were identified in 2–27% of patients without AChR and MuSK antibodies [9,10], and an animal model suggested a pathogenic role for these antibodies [11].

While the underlying causes are not yet determined, there seem to be remarkable ethnic and regional differences in the frequency and clinical features of seronegative MG. For example, in contrast to the relatively uniform frequency of AChR-MG, the incidence of MuSK-MG varies considerably in different regions with an inverse correlation to geographic latitude in Europe and North America [12]. The positive rate of MuSK antibody was also reported to be significantly higher in African-Americans than in Caucasians [13]. In addition, a large frequency variation was noted for LRP4-MG, ranging from 7 to 33% in patients with double seronegative (AChR/MuSK) MG in Europe [14]. A recent study in a British cohort also reported ethnic difference in positive rates of clustered AChR antibodies with a high proportion of non-Caucasians, especially black individuals [6]. Ethnic and regional differences may arise from variations in genetic predisposition and environmental exposure, which suggest the need for further research in this area and possibly different approaches in the diagnosis of seronegative MG. However, serological tests based on novel assays and recently identified antigens are not available for routine clinical practice in many regions where the overall frequency and features of seronegative MG according to antibody have not been determined. Thus, we performed a multi-center study to investigate the clinical features and comprehensive autoantibody profiles, including antibodies to MuSK, LRP4, and clustered AChR, in adult patients seronegative for AChR antibodies by conventional RIPA in South Korea.

## Materials and methods

### Patients

This was a retrospective cross-sectional multi-center study. Clinical data and sera of adult patients with a high index of suspicion for seronegative generalized MG were collected from 18 hospitals between January 2014 and May 2016. Data were entered into a standard case report form designed to record the clinical characteristics of patients with seronegative generalized MG. MG was diagnosed based on the presence of exertional muscle weakness and an abnormal decremental response to low-frequency repetitive nerve stimulation (RNST), or positive pharmacological tests (amelioration of symptoms after intravenous or intramuscular administration of anti-cholinesterase). AChR antibodies should be negative on RIPA. Collected data were reviewed and assessed for inclusion by two authors (KH Park, YH Hong) who had access to all the clinical and laboratory data, including disease course and therapeutic response. Disease severity was evaluated by the Myasthenia Gravis Foundation of America (MGFA) clinical classification, and the MG composite scale (MGCS) [15,16]. All patients provided written informed consent. Sera were stored at -80°C at the central laboratory of Boramae Medical Center, Seoul, before batch transport to the UK for testing. This study was approved by the local ethics committee of Seoul National University, Seoul Metropolitan Government Boramae Medical Center (IRB 16-2014-29).

### Antibody testing

All serum samples were tested for the three autoantibodies to MuSK, LRP4, and clustered AChR at the Autoimmune Neurology Diagnostic Laboratory, Nuffield Department of Clinical Neurosciences, John Radcliffe Hospital, Oxford, UK. All antibody testing was performed blinded to the clinical information. Antibodies to MuSK were tested by both RIPA (RSR Ltd, Cardiff, UK) and CBA, and antibodies to clustered AChR and LRP4 were tested by CBA. Measurement of antibody binding in CBA was performed by indirect immunofluorescence, as previously described [3,6,17]. Results were measured by two observers on a nonlinear visual scale from 0 to 4 with the mean result given. A score of less than 1 was considered to be negative and scores from 1 to 4 were considered to be positive with 1 considered to be weak positive and 4 strong positive. All positive tests were repeated.

### Statistical analysis

Differences between groups were assessed by Fisher’s exact test or Kruskal-Wallis test. Pearson’s correlation analysis was performed to determine the relationship between CBA scores and radioimmunoassay (RIA) values for anti-MuSK antibodies. A two-tailed *P* value < 0.05 was considered to be statistically significant. All statistical analyses were performed with SPSS for Windows version 21.0 (IBM Corp., Armonk, NY).

## Results

### Autoantibody profiles

**Fig 1:**
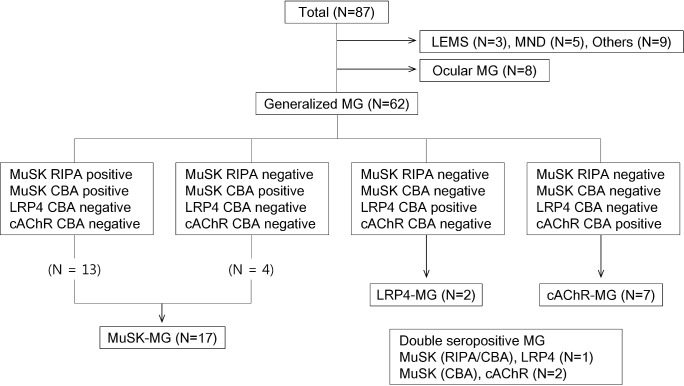
Antibody profiles for MuSK, LRP4, and clustered AChR in patients with MG seronegative for AChR antibody on radioimmunoprecipitation assay. LEMS, Lambert-Eaton myasthenic syndrome; MND, motor neuron disease; MG, myasthenia gravis; MuSK, muscle-specific tyrosine kinase; CBA, cell-based assay; RIPA, radioimmunoprecipitation assay; LRP4, low-density lipoprotein receptor-related protein 4; cAChR, clustered acetylcholine receptor.

**Fig 2:**
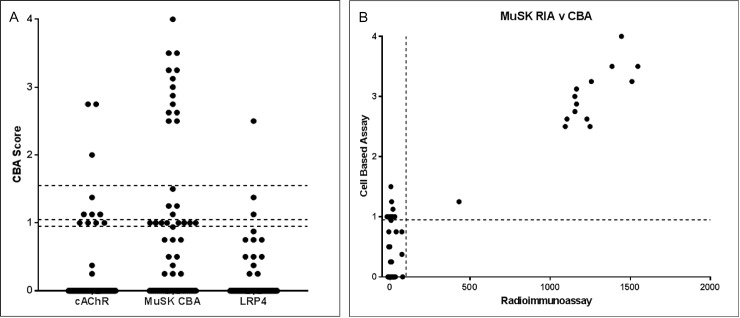
**Results of CBA for antibodies to MuSK, LRP4, and clustered AChR (A) and correlation between CBA scores and RIA values for anti-MuSK antibodies (B)** MuSK, muscle-specific tyrosine kinase; CBA, cell-based assay; LRP4, low-density lipoprotein receptor-related protein 4; AChR, acetylcholine receptor; RIA, radioimmunoassay.

**Fig 3:**
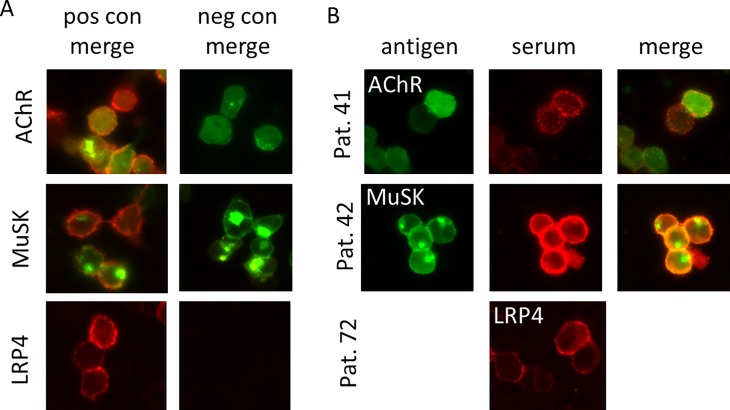
Cell based assays to detect antibodies to clustered AChR, MuSK or LRP4. **Patient IgG binding is shown in red. For the AChR and MuSK assays, EGFP tags exhibit a green fluorescence highlighting the transfected cells; there is no color tag in the LRP4 assay. Patient 41 is positive for AChR, Patient 42 is positive for MuSK antibodies, and patient 72 is low positive for LRP4 antibodies.** MuSK, muscle-specific tyrosine kinase; LRP4, low-density lipoprotein receptor-related protein 4; AChR, acetylcholine receptor.

In total, 87 patients were included in the study. Sixty-two patients had a final diagnosis of generalized MG, 8 of ocular MG, 3 of Lambert-Eaton myasthenic syndrome, 5 of motor neuron disease, and 9 of other or uncertain diagnoses. The results of the antibody tests in 95 serum samples obtained from 87 patients are illustrated in Figs 1 and 2. Of the 62 patients with generalized MG, MuSK antibodies were positive in 17 patients (27%) on CBA and 13 patients (21%) on RIPA. All RIPA-positive samples were positive on CBA. There was a significant correlation between RIPA values and CBA scores for MuSK antibodies (Fig 2). Nine (12.6%) patients were positive for clustered AChR antibodies, and only 2 (2.9%) patients were positive for LRP4 antibodies. Of the 8 patients with ocular MG, 6 were triple seronegative (AChR/MuSK/LRP4) and 2 patients were positive only to clustered AChR. Three (4.3%) patients were double seropositive: one for MuSK (positive in both RIPA and CBA) and LRP4 and two for MuSK (positive in CBA, but negative in RIPA) and clustered AChR. In 5 patients (4 with motor neuron disease and 1 with uncertain diagnosis), the antibody to MuSK was borderline on CBA. Overall, 39 of 70 (55.7%) patients with MG remained triple seronegative for the antibodies to AChR (including clustered AChR), MuSK, and LRP4. Representative images of positive CBA for clustered AChR, MuSK, and LRP4 antibodies are presented in Fig 3.

### Clinical features according to autoantibody profiles

Clinical features of AChR antibody seronegative (on RIPA) generalized MG were summarized and compared between groups classified according to antibody profiles (Table 1). The patients with the LRP4 antibody were not included in the comparison due to their small number. Among the three subgroups (MuSK, clustered AChR, and triple seronegative), significant differences were noted in sex and current use of acetylcholinesterase inhibitor (p < 0.05). There was also a marginally significant difference in the current MGFA classification (proportion of bulbar predominant type). Patients with the MuSK antibody were mostly female (88.2%), had predominantly the bulbar type (70%), and were less likely to be receiving acetylcholinesterase inhibitor treatment. Thymic hyperplasia and thymoma were rare (6.3% and none, respectively), and more than half of the patients had myasthenic crises. In contrast, the patients with antibodies to clustered AChR tended to have mild phenotypes, frequent ocular form at presentation (29%), and good prognosis with higher remission (67%) and lower rates of crisis (33%). There was no significant correlation between MGCS and RIPA titer (r = 0.509, p = 0.091) or CBA score (r = 0.319, p = 0.183).

**Table 1:** Clinical features of AChR antibody seronegative generalized MG (on radioimmunoprecipitation assay) according to the antibody profile.

	MuSK (*n* = 17)	Clustered AChR (*n* = 7)	Triple* Seronegative (*n* = 30)	*P*-values
Female	15 (88.2%)	4 (57.1%)	14 (46.7%)	0.01
Onset age (years)	43.5 (29.5–59.0)	40.0 (26.0–50.0)	42.5 (32.0–54.0)	0.72
Ocular MG at presentation	2 (11.8%)	2 (28.6%)	5 (16.7%)	0.60
MGFA classification at presentation				
≥III	4 (25.0%)	0 (0.0%)	5 (16.7%)	0.33
B classification	10 (71.4%)	3 (60.0%)	11 (44.0%)	0.24
Current MGFA classification**				
≥III	2 (16.7%)	1 (33.3%)	4 (19.0%)	0.80
B classification	7 (70.0%)	0 (0.0%)	4 (28.6%)	0.05
Thymic hyperplasia***	1 (6.3%)	1 (16.7%)	4 (16.0%)	0.62
Thymoma	0 (0.0%)	1 (16.7%)	1 (4.3%)	0.22
RNST (abnormal decrements)	11 (64.7%)	5 (83.3%)	20 (66.7%)	0.68
Current MGCS**	4.5 (2.0–16.0)	9.0 (8.0–17.0)	5.0 (2.0–9.0)	0.25
Current medication**				
Pyridostigmine	3 (25.0%)	3 (100.0%)	12 (57.1%)	0.04
Steroids	5 (41.7%)	3 (100.0%)	15 (71.4%)	0.09
Other ISA	6 (50.0%)	2 (66.7%)	12 (57.1%)	0.85
Thymectomy**	1 (8.3%)	0 (0.0%)	2 (9.5%)	0.85
Remission and MM**	5 (41.7%)	2 (66.7%)	8 (40.0%)	0.68
CSR	2 (16.7%)	0 (0.0%)	1 (5.3%)	0.47
PR	2 (16.7%)	1 (33.3%)	5 (25.0%)	0.77
MM	1 (11.1%)	1 (33.3%)	5 (26.3%)	0.46
Crisis**	7 (58.3%)	1 (33.3%)	6 (28.6%)	0.23

Values are number (%) or median (range).

AChR, acetylcholine receptor; ISA, immunosuppressive agents, MG, myasthenia gravis; MuSK, muscle-specific tyrosine kinase; cAChR, clustered acetylcholine receptor; MGFA, Myasthenia Gravis Foundation of America; B, bulbar; RNST, repetitive nerve stimulation test; MGCS, myasthenia gravis composite scale; MM, minimal manifestation; CSR, complete stable remission; PR, pharmacologic remission.

* Seronegative for the antibodies to AChR (including clustered AChR), MuSK, and LRP4.

** Evaluated only in those patients with follow-up duration ≥ 12 months.

*** On the chest computed tomography.

The clinical features of the patients with the LRP4 antibody and of those who were double seropositive are summarized in Table 2. The two patients with anti-LRP4 (Pts. 1 and 2) had late-onset generalized MG with small cell lung cancer (Pt. 1), and thymoma (Pt. 2). In the first patient, no abnormal increment was noted in the high frequency RNST test and the antibody to the voltage-gated potassium channel was negative. Three patients were seropositive for more than one antibody, one for MuSK and LRP4 (Pt. 3), and the other two for MuSK and clustered AChR (Pts. 4 and 5). The patient with antibodies to both MuSK and LRP4 had presented with only ocular symptoms and later developed bulbar symptoms. Thymic hyperplasia was noted on chest computed tomography, and no myasthenic crisis had occurred during 2 years of follow-up. Patients with antibodies to MuSK and clustered AChR had presented with bulbar (Pt. 4) or limb (Pt. 5) weakness and had achieved remission.

**Table 2:** Clinical features of the patients with the LRP4 antibody, and of the patients who were double seropositive.

Pt no.	Sex/Age	Follow-up duration (months)	Autoantibodies*	MGFA at entry	MGFA current	RNST	Thymus	Current treatment	Crisis	Remission
1	M/57	31	LRP4 (1.125)	IIIa	IIa	Dec	NL	ACEI, PD, TAC	(-)	(-)
2	F/78	159	LRP4 (1.375)	IIIb	IIb	Dec	Thymoma	ACEI, CYP	(-)	(-)
3	F/57	22	MuSK (3), LRP4 (2.5)	I	MM	Dec	Hyperplasia	(-)	(-)	MM
4	M/54	15	MuSK (1), clustered AChR (1.125)	IIIb	MM	Dec	NL	ACEI, AZA	(-)	MM
5	M/74	76	MuSK (1.5), clustered AChR (1.125)	IIa	PR	Dec	NL	TAC	(-)	PR

* Numbers in parentheses were the mean cell-based assay scores measured independently by two observers.

MuSK: muscle-specific tyrosine kinase; AChR: acetylcholine receptor; LRP4: low-density lipoprotein receptor-related protein 4; CBA: cell-based assay; MGFA: Myasthenia Gravis Foundation of America; RNST: repetitive nerve stimulation test; Dec: abnormal decrements > = 10%, NL: normal; ACEI: acetylcholinesterase inhibitors: pyridostigmine; PD: prednisolone; TAC: tacrolimus; AZA: azathioprine; CYP: cyclophosphamide; MM: minimal manifestation; PR: pharmacologic remission; CSR: complete stable remission

## Discussion

Using comprehensive serological tests, we investigated the autoantibody profiles and clinical features of 70 Korean patients with MG seronegative for AChR on conventional assay. Antibodies to MuSK were present in 27% of 62 patients with seronegative generalized MG. The clinical features of the patients with the MuSK antibody were similar to those reported in previous studies, including female predominance, frequent bulbar involvement, rare thymic pathology, intolerance to acetylcholinesterase inhibitor treatment, and poor prognosis with frequent myasthenic crises. Antibodies to clustered AChR were positive in 12.9% of patients (7 generalized and 2 ocular MG), and the patients tended to have a mild phenotype at presentation and good prognosis. LRP4 MG was rare, with the antibody detected in only 3.2% of patients with seronegative generalized MG and in none of the patients with ocular MG.

Overall, antibodies to clustered AChR, MuSK or LRP4 were identified in 25 of 62 (55.7%) generalized MG patients who were seronegative for AChR on conventional assay. The proportion of triple seronegative MG was higher than expected, reaching 45% despite the comprehensive antibody tests. First, this may be attributed to the low positive rates of antibodies of LRP4 and clustered AChR in our patients. As for the positive rate of anti-MuSK, it is similar to those reported in studies of Korean and Japanese cohorts [18,19]. Second, it could be related to the difficulty in diagnosing seronegative MG. We included patients with a high index of suspicion for generalized MG, but the diagnosis of seronegative MG is often difficult in practice because of atypical clinical and laboratory features. Despite the efforts to confirm the diagnosis, we could not exclude the possibility of inclusion of patients who did not have MG. Last, other autoantibodies against agrin, cortactin, and titin were not evaluated [20–23], and further studies on these autoantibodies could decrease the proportion of true seronegative MG.

CBA for MuSK antibodies confirmed the results of conventional RIPA with a significant correlation between CBA scores and RIPA values. Of note, CBA was also positive in 4 additional patients who were RIPA negative, confirming the higher sensitivity of the assay. The higher sensitivity, however, was achieved at the expense of specificity. Indeed, the antibody to MuSK was weak positive with the CBA score ranging from 1 to 1.25 in 5 of 17 patients who had not MG in the present study. The specificity issue of MuSK CBA has been raised in patients with other neuroimmune diseases and healthy controls [24,25]. Given the overlapping clinical features between MuSK-MG and bulbar-onset motor neuron disease, our results suggest that the borderline positivity in conventional CBA should be carefully interpreted in the clinical context.

It has been reported that the concentrations of anti-MuSK correlate with disease severity [26]. It also has been suggested that changes in the antibody concentrations over time could reflect disease activity [27]. Our study did not have a longitudinal design, so we could not examine the correlation between the concentrations of anti-MuSK and disease activity within individuals. As for the analysis across the patients, we could not find any statistically significant correlation between the concentrations of anti-MuSK and severity of symptoms measured with MG composite scores, which may be attributed to the relatively small number of patients. A large-scale longitudinal study is needed to evaluate the potential of anti-MuSK as a biomarker reflecting disease activity.

Previous studies on the antibody to clustered AChR have reported a wide range of positive rates, from 16% to 60% [3–5]. It was relatively low in our patients, accounting for only 12.9% (9/70) of patients with seronegative MG. This might be explained by the fact that we included only adult patients. Rodriguez et al. reported a high proportion of prepubertal onset of MG (66.6%) in the group of patients with antibodies to clustered AChR [6]. In addition, the positive rate of clustered AChR antibody seems to depend on the proportion of ocular MG [4,6]. We had initially designed the present study to include only patients with generalized seronegative MG. This may have led to the relatively low proportion of ocular MG in our cohort with only 8 patients with ocular MG (11.4%) included. Last, it is likely that there may be ethnic or regional differences in the prevalence of seronegative MG with antibody only to clustered AChR. This should be investigated further, particularly in patient cohorts with childhood/juvenile onset and ocular MG.

The frequency of LRP4-MG was very low in the present study with only 5% (2/40) of patients with double seronegative (AChR/MuSK) MG. Previous studies in Japanese and Chinese cohorts also reported very low prevalence of LRP4-MG with 2.2% (6/272) and 1% (2/50), respectively [9,28]. Higher positive rates were reported in Germany (53.8%, 7/13), Italy (14.5%, 8/55), and a multi-national European cohort (18.7%, 119/635) [14,29,30], and an intermediate positive rate (9.2%, 11/120) in a combination of Greek and USA cohorts [10]. These results strongly support ethnic and/or regional differences regarding the frequency of LRP4-MG, with much lower prevalence in East Asian populations than in Western populations. While much remains unknown, it is assumed that genetic and environmental factors are involved. Different methods for antibody testing may affect the difference, but its influence seems to be small. Both German and Chinese cohort studies used CBA, similar to us, but the former reported the highest positive rate, and the latter the lowest rate [28,29]. The Japanese and Italian studies used the same luciferase-reporter immunoprecipitation assay, but reported remarkably different positive rates [9,30].

Although the clinical features of LRP4-MG have not yet been fully defined, the patients were reported to have milder symptoms at onset and favorable prognosis compared to the MuSK- as well as the AChR-MG patient groups [14]. Intriguingly, the clinical features of the two LRP4 antibody-positive patients in our cohort appear to be different from those previously reported. One patient had thymoma, which is an unusual finding in LRP4-MG, just as in MuSK-MG. Furthermore, there was comorbidity of MG and small cell lung cancer in the other patient. There is a limited number of lung cancer cases presenting with MG [31]. According to a recent case series reporting on the very uncommon association between MG and lung cancer, seronegative MG was more frequently found in small cell lung cancer, in contrast to AChR antibody-positive MG that is associated with non-small cell lung cancer [31]. It has also been shown that LRP4 is expressed on multiple tissues, including the lung and immune system organs in humans (The Human Protein Atlas, www.proteinatlas.org). The significance of the association between LRP4-MG and neoplasms, including thymoma and lung cancer remains to be investigated.

Interestingly, we identified double positive sera in three patients: one for MuSK and LRP4 antibodies, and two for MuSK and clustered AChR antibodies. Double seropositivity has been reported in a limited number of patients, and was proposed as a marker for disease severity [14,32–34]. However, the severity of symptoms varied at presentation in our double-seropositive patients. None of the patients suffered a myasthenic crisis, and all patients achieved either minimal manifestation or remission, suggesting that double seropositivity may not necessarily correlate with disease severity and poor outcome.
