# Correction: Myasthenia gravis seronegative for acetylcholine receptor antibodies in South Korea: Autoantibody profiles and clinical features

**DOI:** 10.1371/journal.pone.0200225

**Published:** 2018-06-29

**Authors:** Kee Hong Park, Patrick Waters, Mark Woodhall, Bethan Lang, Thomas Smith, Jung-Joon Sung, Kwang-Kuk Kim, Young-Min Lim, Jee-Eun Kim, Byung-Jo Kim, Jin-Sung Park, Jeong-Geon Lim, Dae-Seong Kim, Ohyun Kwon, Eun Hee Sohn, Jong Seok Bae, Byung-Nam Yoon, Nam-Hee Kim, Suk-Won Ahn, Jeeyoung Oh, Hyung Jun Park, Kyong Jin Shin, Yoon-Ho Hong

In the Funding section, the grant number from the funder Basic Science Research Program through the National Research Foundation of Korea funded by the Ministry of Education is listed incorrectly. The correct grant number is: 2017R1D1A1B03030569.
